# Dynamic contrast enhanced CT in nodule characterization: How we review and report

**DOI:** 10.1186/s40644-016-0074-4

**Published:** 2016-07-18

**Authors:** Nagmi R. Qureshi, Andrew Shah, Rosemary J. Eaton, Ken Miles, Fiona J. Gilbert, on behalf of the Sputnik investigators

**Affiliations:** Department of Radiology, Papworth Hospital, Cambridge, CB23 3RE UK; Department of Radiation Protection, Mount Vernon Hospital, East and North Hertfordshire NHS Trust, Northwood, HA6 2RN UK; Department of Diagnostic Imaging, Princess Alexandra Hospital, Woolloongabba, Brisbane, Australia; Institute of Nuclear Medicine, University College London, London, UK; Department of Radiology, University of Cambridge School of Clinical Medicine, Box 218 Cambridge Biomedical Campus, Cambridge, CB2 0QQ UK

**Keywords:** Dynamic contrast enhanced CT, Solitary pulmonary nodules

## Abstract

Incidental indeterminate solitary pulmonary nodules (SPN) that measure less than 3 cm in size are an increasingly common finding on computed tomography (CT) worldwide. Once identified there are a number of imaging strategies that can be performed to help with nodule characterization. These include interval CT, dynamic contrast enhanced computed tomography (DCE-CT), ^18^F-fluorodeoxyglucose positron emission tomography-computed tomography (^18^F-FDG-PET-CT). To date the most cost effective and efficient non-invasive test or combination of tests for optimal nodule characterization has yet to be determined.

DCE-CT is a functional test that involves the acquisition of a dynamic series of images of a nodule before and following the administration of intravenous iodinated contrast medium. This article provides an overview of the current indications and limitations of DCE- CT in nodule characterization and a systematic approach to how to perform, analyse and interpret a DCE-CT scan.

**Electronic supplementary material:**

The online version of this article (doi:10.1186/s40644-016-0074-4) contains supplementary material, which is available to authorized users.

## Background

Incidental indeterminate solitary pulmonary nodules (SPN) that measure less than 3 cm in size are an increasingly common finding on computed tomography (CT) worldwide. Once identified there are a number of imaging strategies that can be performed to help with nodule characterization. The Fleischner Society clinical guidelines which are widely used recommend performing either 2 year nodule follow up with interval CT, dynamic contrast enhanced computed tomography (DCE-CT), ^18^F-fluorodeoxyglucose positron emission tomography-computed tomography (^18^F-FDG-PET-CT) and/or biopsy for nodules over 8mm [1].

So far the most cost effective and efficient non-invasive test or combination of tests for optimal nodule characterization has yet to be determined. This matter will be of increasing importance if a lung cancer-screening programme is introduced in the future

Currently the role, diagnostic accuracy and cost-effectiveness of DCE-CT in the characterization of SPNs compared to standard management strategies (PET-CT, CT nodule follow up) is being investigated in the National Institute of Health Research (NIHR) Health Technology Assessment funded multi-centre SPUtNik trial which should report in 2018 (ISRCTN 30784948).

DCE-CT is a functional test that involves the acquisition of a dynamic series of images of a nodule before and following the administration of intravenous iodinated contrast medium.

Preliminary results from the SPUtNik trial suggest that DCE-CT is a robust technique where problems with data acquisition are limited and seen in 6% of cases. This can occur for a variety of reasons: the nodule measuring less than 8mm in size at the time of examination, patients refusing cannulation, experiencing panic attacks or difficulties with breath holding.

There are essentially two approaches to performing a nodule DCE-CT examination. The first pass technique assesses the initial intravascular passage of contrast medium through the nodule allowing evaluation of various perfusion related parameters. Results from different stand-alone studies have proved promising but, as yet, this technique is still considered a research tool and not routinely available [2, 3]. The second approach is the more established validated DCE-CT technique that is currently available in clinical practice. This determines the maximal mean nodule enhancement measured in terms of Hounsfield Unit (HU), with malignant nodules typically demonstrating higher mean contrast-enhancement reflecting the presence of tumour neo-vascularisation compared to benign nodules [4, 5]. Pooled analysis of 10 DCE-CT studies (1167 nodules) reported sensitivity, specificity and area under the ROC curve of 93 %, 76 % and 0.93 respectively, which was comparable with the diagnostic performance of FDG PET [6].

Despite these promising results and ease of availability, DCE-CT is not widely used in clinical practice. This seems to be due primarily to the lack of standardized acquisition protocols, post-processing and reporting strategies available. Table 1

**Table 1:** gives the indications, strengths and limitations of the DCE-CT technique

**Fig. 1:**
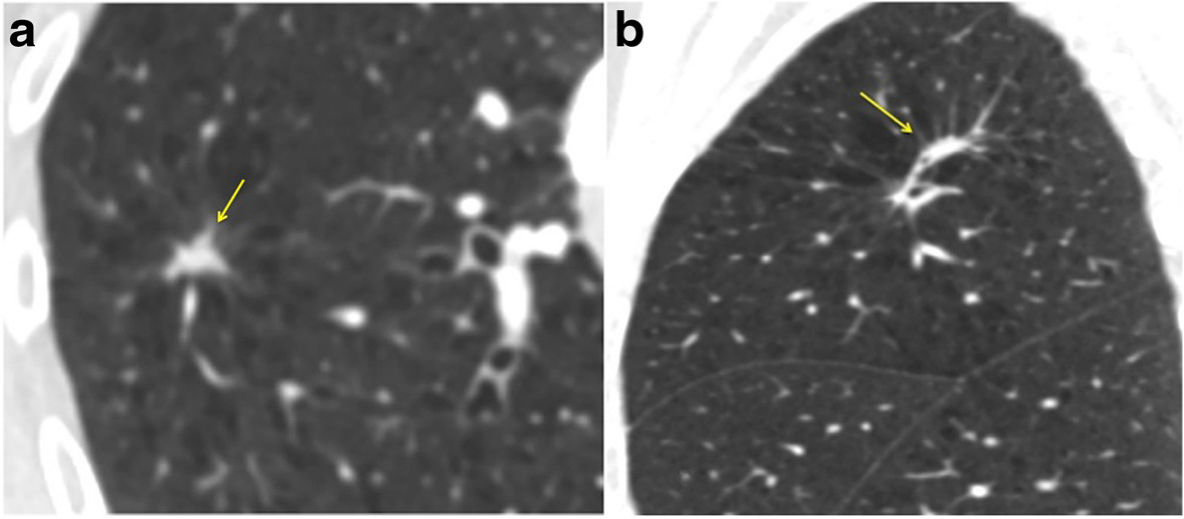
**a** Axial CT shows a possible spiculated 10x8 mm nodule (arrow). **b** On the Coronal reformat the lesion is ‘flat’ and likely represents focal scarring/linear atelectasis and is not suitable for DCE-CT (arrow)

**Fig. 2:**
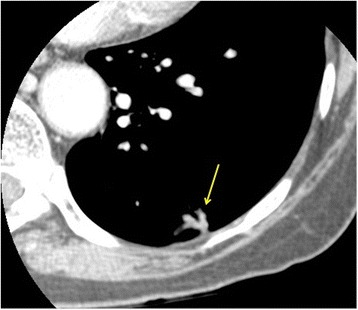
Axial CT shows a ‘polygonal’ nodule with no central spherical component that is also not suitable for DCE-CT

**When should a DCE-CT be performed?** Currently DCE-CT is a complementary imaging test that is usually performed alongside more widely accepted tests such as CT and ^18^F FDG PET-CT to enable further non-invasive lesion characterization. Where in the diagnostic pathway a DCE-CT should ideally be performed during the work up of a SPN is yet to be determined. DCE-CT should be considered for patients with soft tissue SPN that are considered low to moderate risk for malignancy or following a t^18^F FDG PET-CT that shows indeterminate low grade tracer uptake less than the mediastinal blood pool where obtaining tissue diagnosis by biopsy would be considered high risk [1, 7]. DCE-CT is unhelpful in the characterization of pure ground glass nodules and part solid nodules with a soft tissue component that measures < 8 mm. It is also of limited accuracy in ‘flat’ nodules that may measure 8 mm in the axial dimension but only 3-4 mm in the cranio-caudal direction (Fig. 1). Polygonal shaped lesions with no central spherical/oval component where a reproducible region of interest (ROI) cannot be drawn should also be excluded (Fig. 2). As the technique involves the administration of iodinated contrast it is contraindicated in patients with known intravenous contrast allergies and renal impairment. **Strengths and weaknesses of DCE CT.** The main strengths of DCE-CT are the relatively low cost, convenience, the simplicity of image acquisition and the widespread availability of suitable CT machines capable of undertaking the protocol. The examination has a room time of 10mins and the image analysis is straightforward and can be performed on any standard commercial software in a matter of minutes. The disadvantage of this technique is the use of ionizing radiation. With the advent of multi-detector CT previous limitations of reduced anatomic coverage of CT has improved with cranio-caudal coverage of large volumes to encompass a SPN now possible.	

### Protocol

As with any x-ray imaging technique, the optimal approach is usually dependent on achieving a balance between image quality and radiation dose. The DCE-CT technique used in the SPUtNik trial has been determined by comparative measurements varying the current or voltage by weight in a thorax phantom in Mount Vernon Medical Physics Department. For this protocol the signal to noise ratio was chosen as an image quality metric and compared to work published by previous studies [4, 8]. At lower tube voltage the CT number of iodine is enhanced as the average x-ray energy gets closer to the Iodine K-edge, giving better contrast in the image. Therefore, 100kVp was chosen as a balance between increased signal to noise ratio and moderate scan time and scanner power requirements. The chosen tube current aims to maintain image quality for different sized patients. The automated tube current modulation feature on modern scanners is not used as the performance varies from one manufacturer to the next.

The dose selected for this protocol is higher than a standard chest CT scan, primarily to reduce the noise in the lesion and give more confidence in the measured CT (HU) number. The higher dose setting should also help reduce the impact of streak artifacts. Iterative reconstruction is not used because both this and tube current modulation are dependent upon the manufacturer and are not available on all CT scanners. Therefore this protocol allows standardized acquisition of comparable and reproducible datasets across all CT manufacturers and platforms.

Prior to performing DCE-CT, the suitability of the SPN for DCE-CT should be assessed by confirming that the nodule is of soft tissue attenuation, visible on a routine or low dose CT and measures ≥ 8mm when viewed on mediastinal windows (Window Width 400HU, Window Level 40HU). The patient weight should be recorded to calculate the volume of contrast to be injected and the mA protocol required.

The DCE-CT technique involves the acquisition of a dynamic series of short spiral acquisitions centered on the SPN with the patient breath-holding following an intravenous bolus of iodinated contrast medium (300 mg/ml) injected at 2ml/sec. The volume of contrast material will be 1.4 ml/kg. The minimum image data set is summarised below in Table 2.

**Table 2:** CT Imaging parameters

Tube voltage	100 KVp,
Tube current	Determined by patient’s weight: <60Kg, 200mAs 60-90Kg, 350 mAs >90kg. 500 mAs
Rotation time	0.5 s or similar depending on scanner
Pitch	1:1 or similar depending on scanner
Field of View	15 cm or similar depending on scanner
Z-direction coverage	At least 60mm
Detector collimation	To be specified for each scanner model. Typically 64 slices
Slice thickness	3.0 mm
Reconstruction interval	2.0 mm or similar depending on scanner
Image time relative to onset of contrast material injection	Pre- contrast, 60s, 120s, 180s and 240s
Reconstruction algorithm	Siemens B30, GE Standard, Toshiba FC13. Iterative reconstruction (if available) to be switched off.

### Image display and analysis

DCE-CT image analysis can be performed on all standard commercial software platforms that are widely available.

Correct window setting is essential for accurate interpretation. The nodule should be analysed on mediastinal windows (Width 400HU, Level 40HU) the axial plane.

- To display the images divide the screens into a 2x3 or 2x2 grid and drag the axial dataset for the unenhanced, 1, 2, 3 and 4 min dataset (in chronological order) into each frame.
- For analysis, starting with the axial unenhanced scan, scroll through the images and identify the slice on which the nodule is of maximum size and closest to the nodules equator. Then magnify the image.
- On the magnified image manually draw a circular, polygonal or freehand ROI depending on the shape of the SPN that encompasses at least 70% of the nodule diameter taking care to exclude large vessels, adipose tissue and the adjacent lung parenchyma all of which will affect the measured CT (HU) number.
- The software tool should automatically calculate the mean, minimum and maximum CT (HU) number for the drawn ROI as well as the ROI size in mm^2^.
- This should then be repeated for the post contrast 1, 2, 3 and 4 min scans taking care to ensure the ROI size and image slices are comparable.
- Functional quantitative analysis of DCE-CT data can be performed by measuring the maximal mean nodule enhancement by subtracting the baseline unenhanced CT mean (HU) number from each subsequent time point CT (HU) number.

A video showing a step-by-step approach is attached (Additional file 1).

### How to determine if a study is technically adequate

A DCE-CT study is considered technically adequate if the following criteria are met.

1. The entire volume of contrast is injected with no evidence of extravasation [4].
2. Image quality is not degraded by artifacts that preclude accurate measurement of the mean CT (HU) number (see artifacts section).
3. There is no significant respiratory mis-registration between time points allowing comparable measurements to be obtained.
4. The nodule must be visible on more than three axial slices to minimize effects from partial volume averaging from the lung parenchyma.

### Image interpretation

Functional nodule interpretation is determined by the kV applied. Typically a pulmonary nodule that demonstrates an overall mean enhancement >20 HU when scanned at 100kV as in the SPUtNiK trial or >15 HU at 120kV is usually suggestive of being malignant whereas nodule enhancement of <20 HU at 100kV and <15HU at 120kV are strongly predictive of benign nodules [4, 8].

**Fig. 3:**
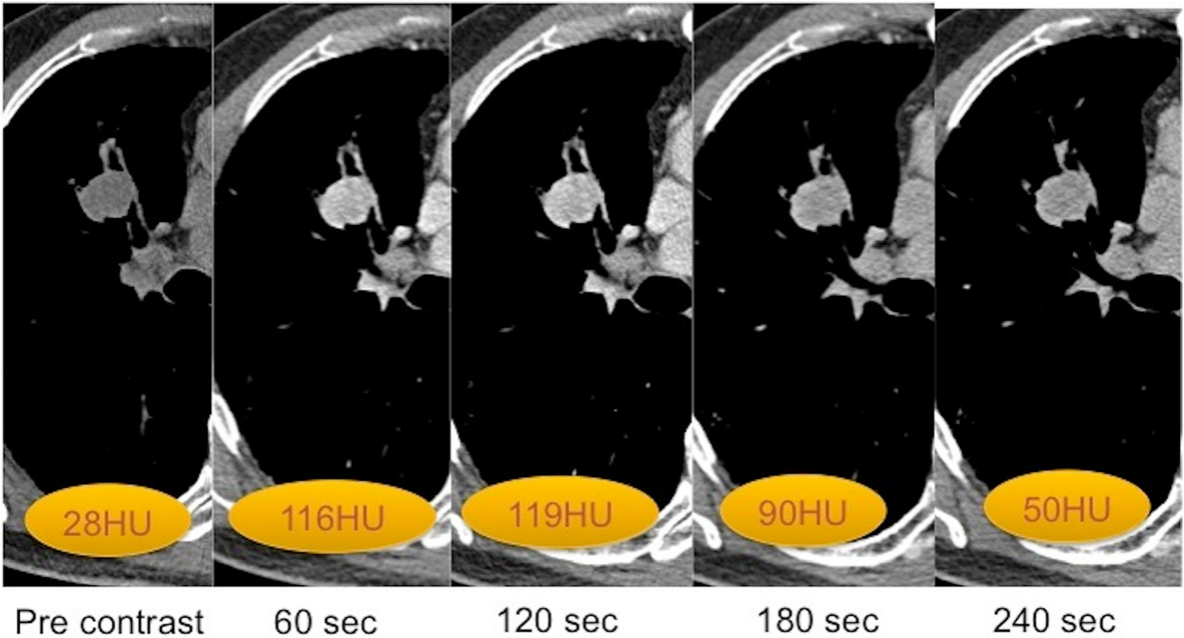
Right upper lobe Carcinoid tumour: Series of axial DCE-CT slices that demonstrates a hyperenhancing lobulated endobronchial nodule with a mean CT enhancement (HU) number 91. CT guided biopsy confirmed a typical carcinoid tumour

Although DCE-CT has high sensitivity in differentiating between malignant and benign lesions it is limited by poor specificity as there is a significant overlap with malignant nodules that show increased nodule enhancement as well as benign processes that result in increased blood flow, perfusion or capillary permeability. This is typically seen with focal organizing pneumonia, acute infective, active inflammatory processes and hypervascular benign pulmonary tumours (Fig. 3). False negatives have also been described with nodules that show central necrosis and limited vascular stroma.

When interpreting DCE-CT it is essential that the mean nodule enhancement is interpreted in the context of the clinical history and the morphological appearances of the nodule. The clinical history should provide information about the pre-test probability for malignancy ideally using the recently published British Thoracic Society guidelines for pulmonary nodules [9]

Morphological appearances can also be helpful as a nodule with a spiculated or irregular margin is more likely to be malignant whereas a nodule with a smooth margin is more likely to be benign, the exception being pulmonary metastases.

### Pitfalls and artifacts in image analysis and interpretation

The main artifacts that can influence the measured CT (HU) number are due to beam hardening, streak artifact, partial volume averaging and cardiac/respiratory motion.

Beam hardening artifact which can arise from adjacent ribs, subcutaneous metallic objects, pacemaker leads and contrast filled cardiovascular structures can lead to additional uncertainty and a lower HU being recorded. Applying beam hardening correction can be unpredictable as sometimes scanners over compensate for the artifact. The measured HU may also be altered by streak artifacts with the location of the streak affecting different slices on each scan. Any slices with streak artifacts across the SPN should therefore be disregarded. Both cardiac and respiratory motion artifact can result in SPN image mis-registration reducing comparable measurements to be obtained. Finally, partial average volume from the adjacent lung parenchyma can also lead to a lower measured HU number particularly when the ROI drawn extends to the edge of the nodule or when the very top/bottom slice of the nodule is chosen for image analysis.

### Formulating a report

Reporting templates have been shown to be extremely useful for improving reporting practices and communication with referrers. This is achieved by providing clear and consistent information presented in a standardized format with a structured easy to follow content and recommendations.

Clinical indication: This should include the nodule’s pre-test likelihood for malignancy. History of previous/current malignancies and smoking history should be included.

Technique: The volume of contrast injected and dose of the DCE-CT scan is recorded. Comment should also be made on the diagnostic quality and adequacy of the study (see earlier section).

Findings: Site (lobe and segment) and size (short and long axis nodule measurements) in mm.

Morphological nodule characteristics: round, well-defined, presence of calcification or macroscopic fat. Lobulated, spiculated or irregular margins.

Functional nodule analysis: including the image series/slice chosen for analysis. Followed by the ROI size and mean HU for each time point and the subsequent calculated maximal mean enhancement value and the time point this was observed.

Recommendations:

Nodules with a maximal mean enhancement >20 HU and:

- High pre-test probability of malignancy will require tissue diagnosis for definitive diagnosis. This can be obtained with a CT guided biopsy, endobronchial ultrasound (EBUS) or surgical frozen section and proceed depending on local practice.
- Low pre-test probability can be monitored with interval low dose CT at 3, 9 and 24 month intervals for a period of 2yrs years.

Nodules with a maximal mean enhancement <20 HU can be followed up with low dose CT at 3, 9 and 24 month intervals for a period of 2 years.

## Conclusions

DCE-CT is currently considered a complementary test to help address limitations in the existing diagnostic work up of indeterminate pulmonary nodules that are clinically considered low to moderate risk for malignancy. This technique has a high negative predictive value for malignancy for nodules that demonstrate <20 HU enhancement. Due to the overlap in enhancement between malignant and hypervascular benign lesions, nodules that enhance >20 HU require tissue diagnosis/follow up for further characteristion.

The multi-center SPUtNIk trial which aims to determine the diagnostic performance, costs and health outcome of incorporating DCE-CT alongside FDG PET-CT will hopefully help further define the role of DCE-CT for nodule characterization.

## Abbreviations

^18^F-FDG-PET-CT, ^18^F-fluorodeoxyglucose positron emission tomography-computed tomography; CT, Computed tomography; DCE-CT, Dynamic contrast enhanced computed tomography; HU, Hounsfield unit; SPN, Solitary pulmonary nodule; SPUtNik trial, ‘Accuracy and cost-effectiveness of dynamic contrast enhanced computed tomography in the characterisation of solitary pulmonary nodules’

## Additional file

Additional file 1: A video showing a step-by-step approach of how to display and analyse a DCE-CT examination. (MP4 37970 kb)

## References

[1] Macmahon H, Austin JH, Gamsu G et-al. Guidelines for management of small pulmonary nodules detected on CT scans: a statement from the Fleischner Society. Radiology. 2005;237(2):395-400. 10.1148/radiol.2372041887 16244247

[2] Ohno Y, Nishio M, Koyama H, Seki S, Tsubakimoto M, Fujisawa Y, Yoshikawa T, Matsumoto S, Sugimura K (2015). Solitary pulmonary nodules: Comparison of dynamic first-pass contrast-enhanced perfusion area-detector CT, dynamic first-pass contrast-enhanced MR imaging, and FDG PET/CT. Radiology.

[3] Yeon Joo Jeong, Kyung Soo Lee, Sun Young Jeong, Myung Jin Chung, Sung Shine Shim, Hojoong Kim, O Jung Kwon, and Seonwoo Kim. Solitary Pulmonary Nodule: Characterization with Combined Wash-in and Washout Features at Dynamic Multi–Detector Row CT. Radiology. 2005;237:2, 675-683. 10.1148/radiol.2372041549 16244276

[4] Stephen J. Swensen, Robert W. Viggiano, David E. Midthun, Nestor L. Müller, Andrew Sherrick, Keiji Yamashita, David P. Naidich, Edward F. Patz, Thomas E. Hartman, John R. Muhm, and Amy L. Weaver. Lung Nodule Enhancement at CT: Multicenter Study. Radiology. 2000;214:1, 73-80. 10.1148/radiology.214.1.r00ja1473 10644104

[5] O’Connor JPB, Tofts PS, Miles KA, Parkes LM, Thompson G, Jackson A (2011). Dynamic contrast-enhanced imaging techniques: CT and MRI. Br J Radiol.

[6] Cronin P, Dwamena BA, Kelly AM, Carlos RC (2008). Solitary pulmonary nodules: meta-analytic comparison of cross-sectional imaging modalities for diagnosis of malignancy. Radiology.

[7] Gould MK, Fletcher J, Iannettoni MD, Lynch WR, Midthun DE, Naidich DP, Ost DE. Evaluation of patients with pulmonary nodules: when is it lung cancer?: ACCP evidence-based clinical practice guidelines (2nd edition). Chest. 2007;132(3 Suppl):108S–30S. 10.1378/chest.07-1353 17873164

[8] Miles KA, Lee TY, Goh V, Klotz E, Cuenod C, Bisdas S, Groves AM, Hayball MP, Alonzi R, Brunner T (2012). Current status and guidelines for the assessment of tumour vascular support with dynamic contrast-enhanced computed tomography. Eur Radiol.

[9] M E J Callister, D R Baldwin, A R Akram, S Barnard, P Cane, J Draffan, K Franks, F Gleeson, R Graham, P Malhotra, M Prokop, K Rodger, M Subesinghe, D Waller, I Woolhouse, British Thoracic Society Pulmonary Nodule Guideline Development Group, on behalf of the British Thoracic Society Standards of Care Committee BTS guidelines:British Thoracic Society guidelines for the investigation and management of pulmonary nodules. Thorax. 2015;70:Suppl 2 ii1-ii54 doi:10.1136. 10.1136/thoraxjnl-2015-207168 26082159

